# Digital Health Dashboards for Decision-Making to Enable Rapid Responses During Public Health Crises: Replicable and Scalable Methodology

**DOI:** 10.2196/46810

**Published:** 2023-06-30

**Authors:** Tarun Reddy Katapally, Sheriff Tolulope Ibrahim

**Affiliations:** 1 Digital Epidemiology and Population Health Laboratory (DEPtH Lab), School of Health Studies Faculty of Health Sciences Western University London, ON Canada; 2 Department of Epidemiology and Biostatistics Schulich School of Medicine Western University London, ON Canada; 3 Lawson Health Research Institute London, ON Canada

**Keywords:** big data, COVID-19, digital citizen science, digital dashboards, digital epidemiology, digital health, digital health platforms, eHealth, health equity, mHealth, pandemics, public health surveillance, virtual health care, mobile phone

## Abstract

**Background:**

The COVID-19 pandemic has reiterated the need for cohesive, collective, and deliberate societal efforts to address inherent inefficiencies in our health systems and overcome decision-making gaps using real-time data analytics. To achieve this, decision makers need independent and secure digital health platforms that engage citizens ethically to obtain big data, analyze and convert big data into real-time evidence, and finally, visualize this evidence to inform rapid decision-making.

**Objective:**

The objective of this study is to develop replicable and scalable jurisdiction-specific digital health dashboards for rapid decision-making to ethically monitor, mitigate, and manage public health crises via systems integration beyond health care.

**Methods:**

The primary approach in the development of the digital health dashboard was the use of global digital citizen science to tackle pandemics like COVID-19. The first step in the development process was to establish an 8-member Citizen Scientist Advisory Council via Digital Epidemiology and Population Health Laboratory’s community partnerships. Based on the consultation with the council, three critical needs of citizens were prioritized: (1) management of household risk of COVID-19, (2) facilitation of food security, and (3) understanding citizen accessibility of public services. Thereafter, a progressive web application (PWA) was developed to provide daily services that address these needs. The big data generated from citizen access to these PWA services are set up to be anonymized, aggregated, and linked to the digital health dashboard for decision-making, that is, the dashboard displays anonymized and aggregated data obtained from citizen devices via the PWA. The digital health dashboard and the PWA are hosted on the Amazon Elastic Compute Cloud server. The digital health dashboard’s interactive statistical navigation was designed using the Microsoft Power Business Intelligence tool, which creates a secure connection with the Amazon Relational Database server to regularly update the visualization of jurisdiction-specific, anonymized, and aggregated data.

**Results:**

The development process resulted in a replicable and scalable digital health dashboard for decision-making. The big data relayed to the dashboard in real time reflect usage of the PWA that provides households the ability to manage their risk of COVID-19, request food when in need, and report difficulties and issues in accessing public services. The dashboard also provides (1) delegated community alert system to manage risks in real time, (2) bidirectional engagement system that allows decision makers to respond to citizen queries, and (3) delegated access that provides enhanced dashboard security.

**Conclusions:**

Digital health dashboards for decision-making can transform public health policy by prioritizing the needs of citizens as well as decision makers to enable rapid decision-making. Digital health dashboards provide decision makers the ability to directly communicate with citizens to mitigate and manage existing and emerging public health crises, a paradigm-changing approach, that is, inverting innovation by prioritizing community needs, and advancing digital health for equity.

**International Registered Report Identifier (IRRID):**

RR1-10.2196/46810

## Introduction

In this digital age, human engagement with and through internet-connected devices generates a substantial amount of big data [1]. The ever-increasing usage of information technology in all spheres of human life has contributed to this phenomenon [2]. The use of these big data, which traditionally exist outside health care systems, has significant implications for the prediction and prevention of population health crises [3]. However, the exponential growth of big data creates challenges to meaningfully extract useful and timely information to inform public health policies [2,4].

Decision-making processes, particularly from the health systems perspective are complex. This complexity is compounded during population health crises, as evidenced by the difficulty that governments and health systems faced during the COVID-19 pandemic [5]. The challenges that the COVID-19 pandemic continues to pose reiterate the need for timely, appropriate, and precise evidence to inform decision-making during the evolving nature of public health crises [6].

The COVID-19 pandemic has revealed the gaps in governmental and health system decision-making while clarifying the need for a transformative change in the way public health crises need to be managed in the future [7]. The challenge in monitoring, managing, and mitigating public health crises, particularly rapidly spreading and evolving communicable disease epidemics and pandemics, is the need to coordinate decision-making processes across local, provincial, national, and international jurisdictions [8].

The COVID-19 pandemic particularly demonstrated the challenges faced by local jurisdictions in implementing federal or state or provincial mandates [9] due to the lack of timely access to jurisdiction-specific evidence [9]. The COVID-19 pandemic has also reiterated the need for cohesive, collective, and deliberate societal efforts to address inherent inefficiencies in our health systems and overcome decision-making gaps using real-time data analytics [10]. Nonetheless, the pandemic has also provided opportunities to decentralize and democratize technology that can support local decision makers with real-time access to big data for timely decision-making [11,12].

However, ethically obtaining, processing, and visualizing big data from citizens is critical to transforming local jurisdictional decision-making processes that impact public health [3,13]. To achieve this, decision makers need independent and secure digital health platforms that engage citizens ethically to obtain big data, analyze and convert big data into real-time evidence, and finally, visualize this evidence to inform rapid decision-making [3,14].

This visualization of data can be realized by the development and implementation of digital health dashboards [15], which are powerful tools to facilitate rapid decision-making [7]. This study enumerates the development of replicable and scalable jurisdiction-specific digital health dashboards for rapid decision-making to ethically monitor, mitigate, and manage public health crises via systems integration, that is, going beyond health systems.

## Methods

### Dashboard Development Process

The development of the digital health dashboard was in response to transform rapid responses to pandemics such as COVID-19 as well as to decentralize technology that can be sustainable, and scalable across jurisdictions. The primary approach in the development of the dashboard was the use of global digital citizen science to tackle pandemics like COVID-19 [3], which identifies the development of decision-making dashboards as a critical advancement in addressing public health crises via real-time data analytics using citizen-driven big data.

The methodology intersects digital citizen science and mHealth to conceptualize and develop digital health platforms that enable ethical surveillance, integrated knowledge translation, and real-time interventions [12]. Such development requires robust theoretical underpinnings such as the Smart Framework [12], which integrates citizen science, community-based participatory research, and systems science through ubiquitous tools to conduct population health interventions in the digital age. Smart Framework operationalizes the repurposing of citizen-owned ubiquitous communication devices (ie, smartphones), an approach that was incorporated into the development of this digital health dashboard. However, beyond the ability to ethically generate big data for rapid responses, this methodology also operationalizes the capacity of smartphones to enable health equity by empowering disenfranchised citizens to inform jurisdictional policies, that is, digital health for equity.

The first step in the development process was to establish a Citizen Scientist Advisory Council using Digital Epidemiology and Population Health Laboratory’s (DEPtH Lab) community partnerships [16]. The diverse and inclusive 8-member council had representation from across varied groups that included gender, ethnicity, and socioeconomic diversity. More importantly, the council consisted of both community members and decision makers, a key component in the development of jurisdictional-specific digital health dashboards for decision-making.

**Figure 1:**
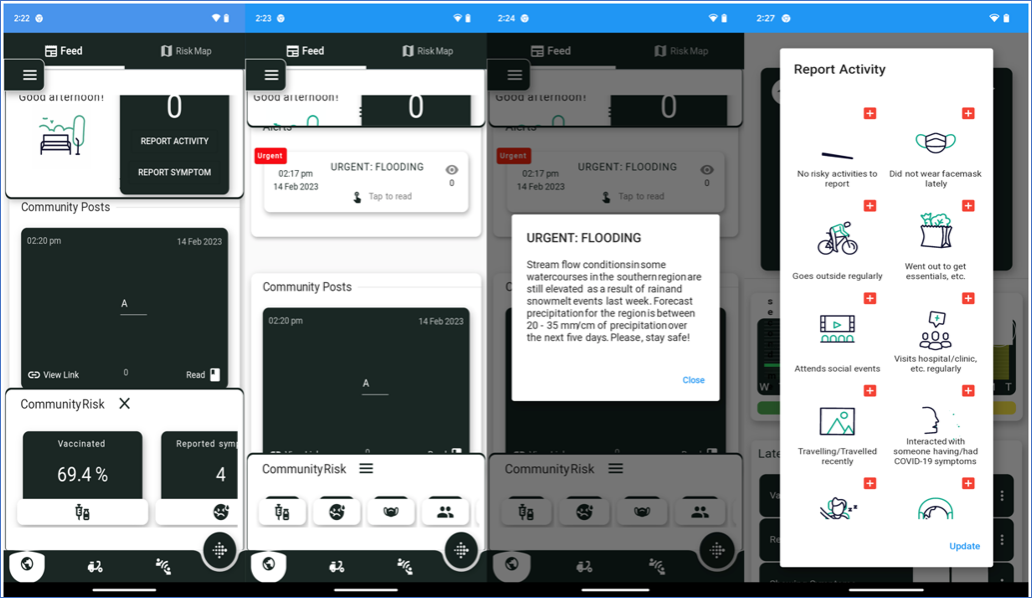
Screenshots of progressive web application.

Based on the consultation with the council, three critical needs of citizens were prioritized: (1) management of household risk of COVID-19, (2) facilitation of food security, and (3) understanding citizen accessibility of public services. Thereafter, a progressive web application (PWA) was developed to provide daily services that address these citizen needs (Figure 1). The big data generated from citizen access to these PWA services are set up to be anonymized, aggregated, and linked to the digital health dashboard for decision-making, that is, the dashboard displays anonymized and aggregated data obtained from citizen devices via the PWA.

The dashboard was developed to achieve the following objectives identified by the global digital citizen science policy [3]: (1) implementation of ethical real-time surveillance to assess community health risks, (2) implementation of near real-time integrated knowledge translation based on the data provided by citizen usage of PWA, (3) implementation of evidence-based real-time public health communication, (4) evidence-based decision-making using real-time data analytics to mitigate health risks, and (5) addressing existing and evolving public health crises by developing adaptable, scalable, and replicable digital infrastructure.

The PWA (Figure 1) serves as a public health advising platform for citizens within a jurisdiction, where community members can manage their household risk of COVID-19 based on an evidence-based algorithm that takes into consideration not only the number of members in a household but also individual and collective household behaviors and symptoms. Using the PWA, citizens are also able to interact with jurisdictional decision makers, request food when in need, and report issues with access to public services within their jurisdiction. The PWA also has the capacity to directly receive alerts from decision makers via the digital health dashboard.

The PWA was designed using gamification features derived from Animaker [17], a software developed by RS Raghavan and coded using Flutter Framework. Flutter is an open-source user interface development kit developed and launched by Google Inc (Mountain View, California) and can be used to develop cross-platform applications for Android, iOS, Linux, macOS, Windows, Google Fuschia, and the web using a single codebase [18,19]. Flutter applications are written in Dart programming language [20].

The PWA is linked to the digital health dashboard through the “hypertext transfer protocol” (http) request and “hypertext preprocessor” (PHP) coding, an open-source server-side scripting programming language, which executes programming instructions at runtime [21]. http and custom-written PHP scripts act as a bridge that relays data from one end to another—PWA to database to Digital Health Dashboard [22]. All functionalities of the PWA are directly linked to the connection made with the backend database, via http request and PHP scripts.

**Figure 2:**
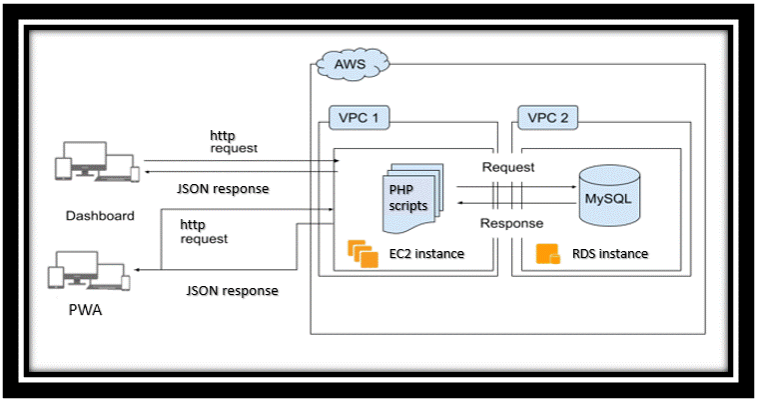
Digital health dashboard data flow processes showing linkage between PWA, dashboard, and cloud storage. AWS: Amazon Web Services; EC2: elastic compute cloud; MySQL: structural query language; PHP: hypertext preprocessor; PWA: progressive web application; RDS: relational database service; VPC: virtual private cloud.

Two virtual private cloud environments, which are hosted on Amazon Web Services (AWS), securely store and communicate data between the database, PWA, and the digital health dashboard (Figure 2) [23]. A virtual private cloud is a private cloud computing environment on shared public cloud infrastructure, which allows for secure computing, networking, and storage of information [24]. Within the virtual private cloud environment, both the Amazon Elastic Compute Cloud servers and the Amazon Relational Database servers are located. Amazon Elastic Compute Cloud servers provide secure and reliable server infrastructure with the flexibility to scale-up or -down server needs [25].

Amazon Elastic Compute Cloud servers host the digital health dashboard through a PHP script run from within the server. Amazon Relational Database Server is a database service provided by AWS that stores, organizes, and makes data available through cloud infrastructure [26]. All data that are being generated through the interaction of citizens with PWA are stored and organized in the Amazon Relational Database using MySQL, an open-source relational database management system [26].

The PHP scripts communicate with the Amazon Elastic Compute servers, and MySQL is used to communicate with the Amazon Relational Database servers to organize incoming data in the database. The activities of the Amazon Elastic Compute Cloud servers and the Amazon Relational Database servers are communicated to both the PWA and digital health dashboard via http [27].

The digital health dashboard is coded and developed in PHP, which allows for the creation of dynamic content on websites to interact with databases [28]. Within the digital health dashboard, there is an interactive community statistical navigation system that was designed and developed using the Microsoft Power Business Intelligence tool [29,30]. Microsoft Power Business Intelligence is a tool that connects data from different sources to provide advanced and interactive data analytics via an automatic data feed to the dashboard. This data gateway ensures near real-time visualization of big data on the dashboard, which can be updated from intervals of 5 to 30 minutes. To ensure the availability of a consistent data feed to the digital health dashboard, the MySQL relational database management system is used to query, access, prepare, and integrate the most recent data points [24].

The dashboard system is set up to anonymously assign QR codes to households within a jurisdiction, which allows the PWA to be used only when QR codes are scanned. Figure 2 presents the complete data flow between the PWA, the Amazon Relational Database, the Amazon Elastic Compute Cloud, and the digital health dashboard. Data at rest are encrypted using randomized keys generated by AWS key management service that gives centralized control over the cryptographic keys used to protect data. All users’ passwords are hashed and stored using Secure Hashing Algorithms [31], which shorten passwords into smaller forms that cannot be understood using bitwise operation, modular additions, or compression functions.

Data in transit are protected by Cloudflare. All media data are stored in Amazon Simple Storage Service [32], which is an object storage service that offers scalability, security, and performance [32]. Media files are moved through AWS CloudFront, a process that prevents users from directly accessing or manipulating Amazon Simple Storage Service [33,34]. Cloud database access is controlled by AWS Identity and Access Management service [35], which helps to securely control access to AWS resources. Finally, all database access processes are audited through AWS CloudTrail [36], which tracks actions taken on AWS accounts [36], an additional security protocol to monitor and prevent data breaches.

### Ethical Considerations

This development process is exempt from ethics review, as study participants were not included in this endeavor. When the digital dashboard will be launched, informed consent will be obtained via the PWA before any data are collected. The information in the informed consent will include the project title, purpose and objectives of the digital health dashboard and PWA, potential benefits and risks, data collection and storage procedures, right to withdraw or drop out and delete the PWA, and contact information for questions, queries, and concerns. Citizens will have to provide informed consent before signing up to use the PWA. All data from citizens will be anonymized and aggregated before data visualization, analyses, and dissemination.

## Results

### Overview of the Developed Dashboard

**Figure 3:**
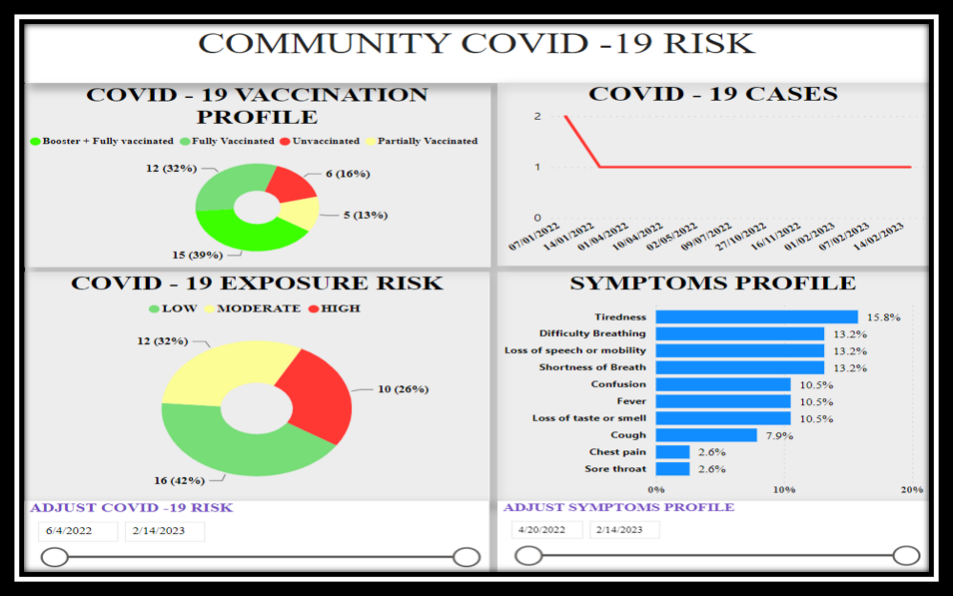
Dashboard of home page displaying community COVID-19 risk profile.

**Figure 4:**
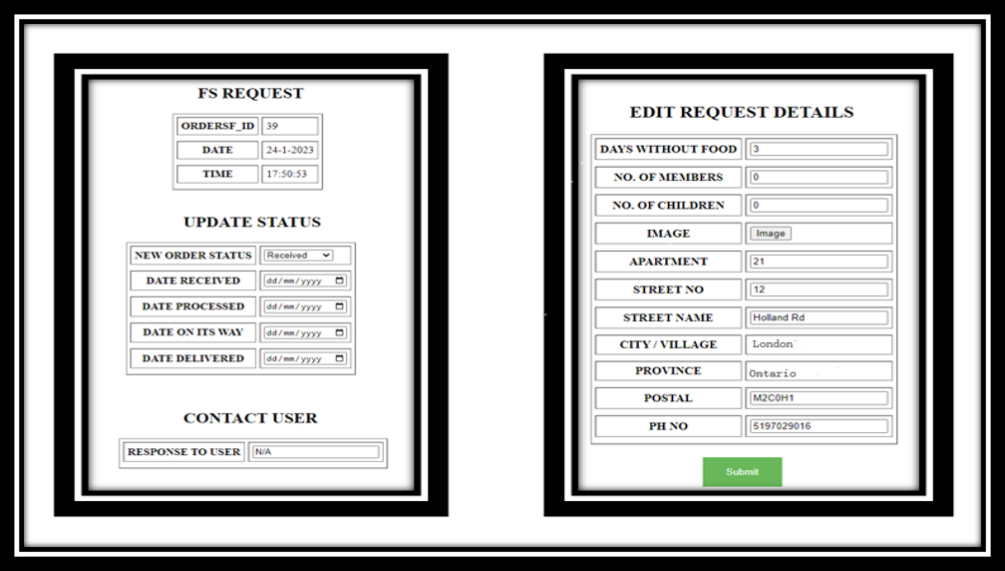
Dashboard of food security status, which allows decision makers to manage food requests. FS: food security.

**Figure 5:**
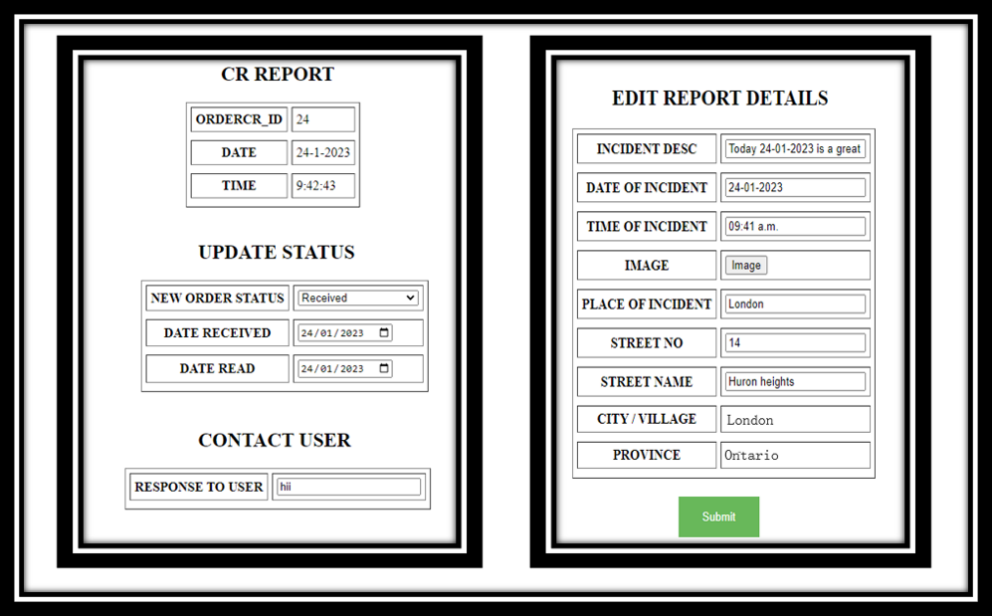
Citizens reporter page, which allows decision makers to view citizen issues in real time. CR: citizen reporter.

**Figure 6:**
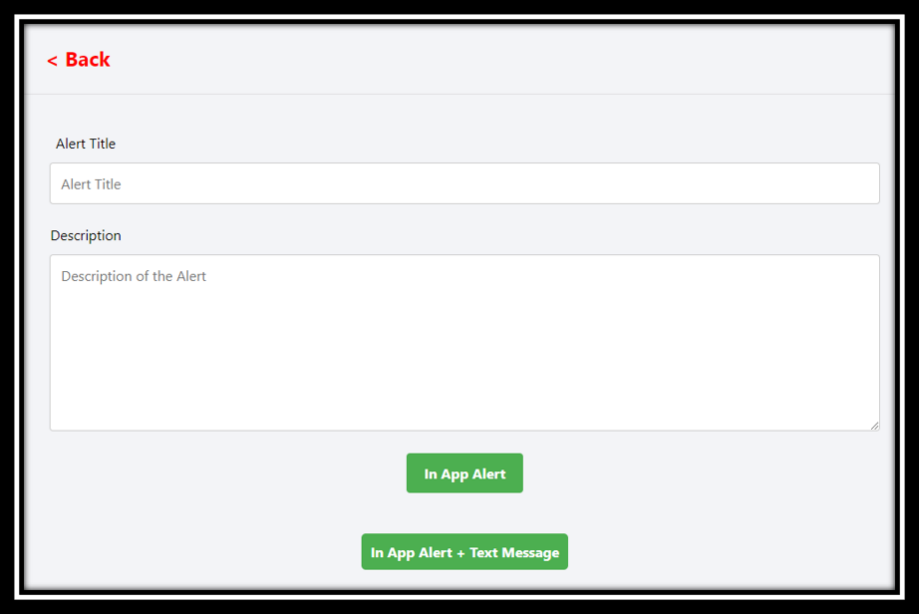
Delegated community alert system, which enables decision makers to send alerts via the direct bidirectional engagement system (Q & A).

**Figure 7:**
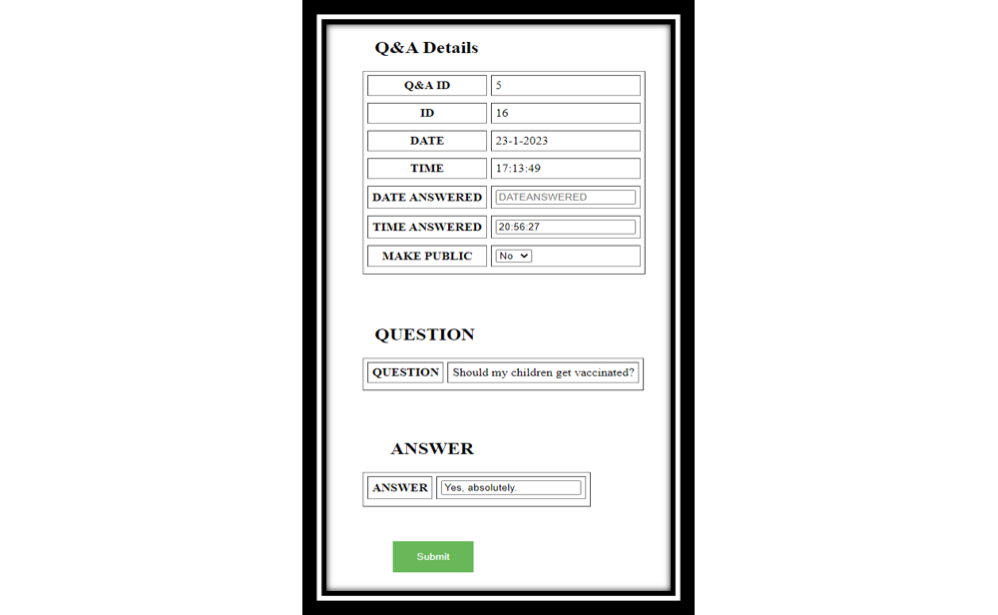
Direct bidirectional engagement system depicting decision makers’ response to anonymous queries.

**Figure 8:**
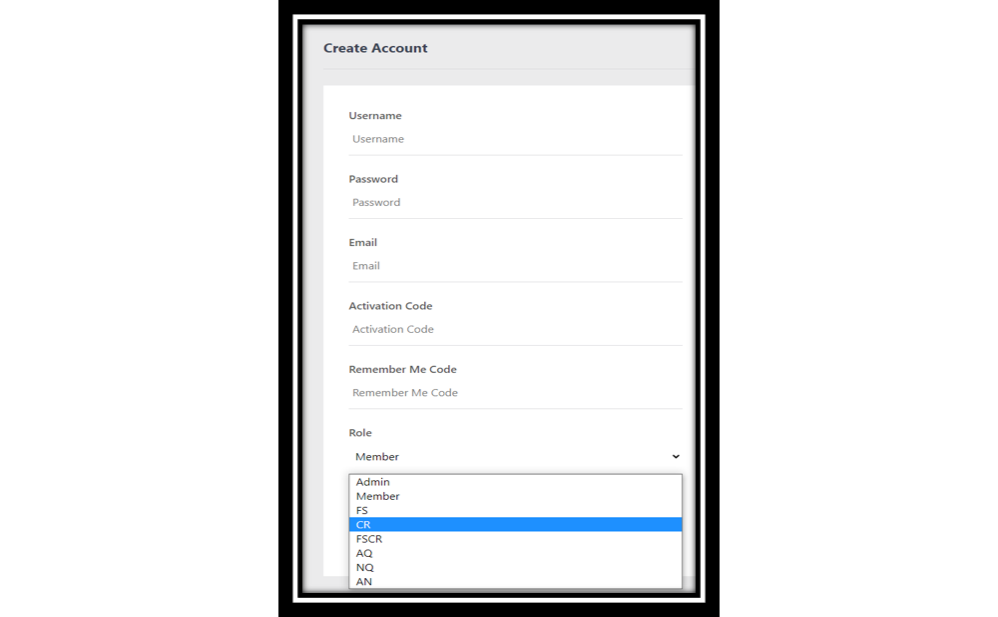
Delegated dashboard access showing categories that can be assigned by the super administrator. AN: alert and community alert; AQ: alert and question and answer; CR: citizen reporter; FS: food security; FSCR: food security and citizen reporter; NQ: community alert and questions and answer.

The development process resulted in a replicable and scalable jurisdiction-specific digital dashboard for decision-making that was tested within the DEPtH Lab to confirm not only the real-time linkage between the PWA and the digital health dashboard but also the functionality of all the resultant features. The big data that are relayed to the dashboard in real time reflect usage of the PWA that provides households the ability to manage their risk of COVID-19, request food when in need, and report difficulties and issues in accessing public services. The main components of the dashboard are (1) community COVID-19 risk profile (Figure 3), (2) food security status (Figure 4), and (3) citizen reporter statistics (Figure 5). However, beyond these core components, there are three additional features of the dashboard: (1) delegated community alert system that enables direct communication with community members (Figure 6), (2) direct bidirectional engagement system (Q & A) that allows decision makers to directly respond to citizen queries (Figure 7), and (3) delegated dashboard access (Figure 8) that provides key decision makers to control and specify staff access to individual components of the dashboard, such as the food security status.

### Community COVID-19 Risk Profile

It depicts the trend lines of COVID-19 cases in a jurisdiction, the COVID-19 vaccination status of the community, which includes the number and proportion of fully (in this case, 2 doses of Health Canada–approved vaccines) and partially vaccinated community members as well as community members who have obtained booster doses. The vaccination profile also depicts the number and proportion of unvaccinated individuals in the community. In addition to this, based on the algorithm developed by the DEPtH Lab, the daily household COVID-19 risk that PWA users obtain at the frontend is anonymized and aggregated before being reflected on the dashboard within the community COVID-19 risk profile. This feature of the dashboard also shows the evolving picture of community symptoms and behavior profile.

### Food Security Status

The food security status feature is a comprehensive decision-making system that enables decision makers to respond to and manage the needs of households in near real time. With a delegated access option, which can be linked with local food banks, the food security status feature provides time-stamped management, displaying the status of the request at various stages: received, processed, dispatched, and delivered. This change of status is reflected in near real time on the PWA, which allows citizens to monitor the progress of their request.

### Citizen Reporter Statistics

This feature reflects time-stamped, and detailed statistics of public services access issues reported by citizens within the jurisdiction, including the description of the issue as detailed by the citizens. The dashboard is set up to respond to the citizens’ report to ensure that there is clear communication between the decision makers and the citizens. For instance, the citizen reporter statistics are further categorized based on the selected time period to show various aspects of the reports, including the number and proportion of acknowledged reports, and the number and proportion of reports yet to be acknowledged. In addition, there are 2 data visualization layers that display the number of reports awaiting a response and number of reports that received a reply.

### Delegated Community Alert System

The decision-making dashboard has a delegated alert system (Figure 6) to send alerts to citizens in the jurisdiction in real time to manage risks, communicate directly, and update citizens of events in the jurisdiction. The delegated level of alerts provides the flexibility to the decision makers to prioritize alerts into urgent and nonurgent alerts. The decision makers have the flexibility to use the alerts from disaster preparation and management to communication of policies.

### Direct Bidirectional Engagement System (Q & A)

To enable anonymized engagement with citizens, a bidirectional engagement system (Figure 7) provides decision makers the ability to address citizen queries and inquiries related to jurisdictional policies and procedures. To provide clarity of communication, the citizens are able to visualize the status of their queries and inquiries at various stages, from “received” and “acknowledged” to “processing” and “responded.” More importantly, the decision makers are able to delegate the queries to ensure that the responses are provided in a timely manner.

### Delegated Dashboard Access

The dashboard is set up to delegate tasks across decision makers, minimize risk of inappropriate access to data, and manage the needs of jurisdictions efficiently by spreading the load of work across several levels of decision makers (Figure 8). For instance, there is a primary “super administrator” who has access to all aspects of the dashboard and has the capacity to delegate access across 7 different categories, which would delineate access to manage specific systems of the dashboard such as “food security” or “citizen reporter.”

### Data Visualization

Finally, data that flow from the AWS Relational Database cloud server are processed and converted into meaningful statistics—a key component that enables all dashboard functions. Through data layering, each visual on the interactive and dynamic dashboard can be explored to ascertain specific and more detailed information about the aggregated visualization. The visualization flexibility allows decision makers to understand patterns that are important for rapid decision-making. For instance, a decision maker might be interested in knowing the demographic distribution of fully vaccinated citizens, which can be visualized by selecting the dropdown options within the dashboard.

## Discussion

### Implications of the Developed Dashboard

During the COVID-19 pandemic, jurisdictions across the world and particularly communities in rural and remote regions faced the need to overcome the decision-making gap that was created due to the lack of timely access to jurisdictional specific information [37]. The pandemic highlighted the inequities between communities, where risk and spread of illness varied due to unequal distribution of adequate health facilities, health care workforce, and health literacy, among other factors [37,38]. The urgency created by the pandemic clarified the need for jurisdiction-specific decision-making technology to address public health crises that prioritizes citizen needs [3]

The pandemic also reiterated the need to decentralize and democratize public health decision-making processes, which would enable rapid responses to public health crises [11,12]. The primary goal of this study was the development of replicable and scalable jurisdiction-specific digital health dashboards for rapid decision-making to ethically monitor, mitigate, and manage public health crises via systems integration, that is, going beyond health systems. The developed dashboard achieves the 5 objectives articulated by the global digital citizen science policy [3].

The community COVID-19 risk profile (Figure 3) feature achieves objective 1 of the dashboard development in implementation of ethical real-time surveillance to assess community health risks [39]. The citizen reporter feature (Figure 5) adds another dimension in achieving objective 1 of dashboard development in obtaining personalized and qualitative perception of citizens in understanding access to public services. Food security feature (Figure 4) achieves objective 2 of the dashboard development in implementation of near real-time integrated knowledge translation based on the information provided by citizens. In visualizing the food security status within the dashboard, decision makers not only understand the aggregated needs of households in the community but also respond to the specific needs of each household requesting food [40].

The delegated alert system (Figure 6) addresses objectives 3 and 4 of the dashboard development: implementation of evidence-based real-time public health communication and evidence-based decision-making using real-time data analytics to mitigate health risks. Bidirectional engagement system (Figure 7) adds personalization in achieving objective 3 of the dashboard development: real-time public health communication [41]. The digital health dashboard in general addresses objective 5 of the dashboard development, that is, development of adaptable, scalable, and replicable digital infrastructure to address existing and evolving public health crises. For instance, using the developed digital health infrastructure, changes can be made to the features of the dashboard to suit jurisdictional, citizen, or crisis-specific needs [40].

More importantly, the digital health dashboard that was developed operationalizes four key processes that have important implications in transforming public health policy in the digital age: (1) evidence-based decision-making, (2) digital citizen science for health equity, (3) rapid responses to public health crises, and (4) social innovation: decentralization of technology, and data sovereignty and security.

### Evidence-Based Decision-Making

Decision-making from the perspective of managing public health crises goes beyond health systems to include key aspects for survival and societal functioning, such as food security [42,43], maintenance of law and order [44,45], and social support to vulnerable groups [46], that is, systems integration for cohesive decision-making. However, making informed decisions in public health crises can be extremely challenging without accurate data and evidence, particularly in situations like pandemics, where disjointed and disparate information from multiple sources can result in poor decision-making [7,47]. This challenge was particularly pronounced in terms of ineffective information sharing across different levels of governments, with municipal governments having to take decisions at the local level, without concrete evidence [48,49].

To overcome these challenges to timely evidence-based decision-making, citizen-driven big data, which is not only timely but also relevant to specific jurisdictions, can transform local jurisdictional decision-making [3,7,49]. The digital health dashboard for decision-making that we conceptualized and developed, at its core, addresses these challenges by providing municipal decision makers access to ethically obtained big data from their own citizens to enable evidence-based decision-making. The decision-making dashboard can not only provide time-stamped big data about a specific public health crisis (ie, COVID-19) but also enables the management of citizen-reported issues that can range from food insecurity to access to public services—a functionality that can ethically monitor the status of community health [50].

**Figure 9:**
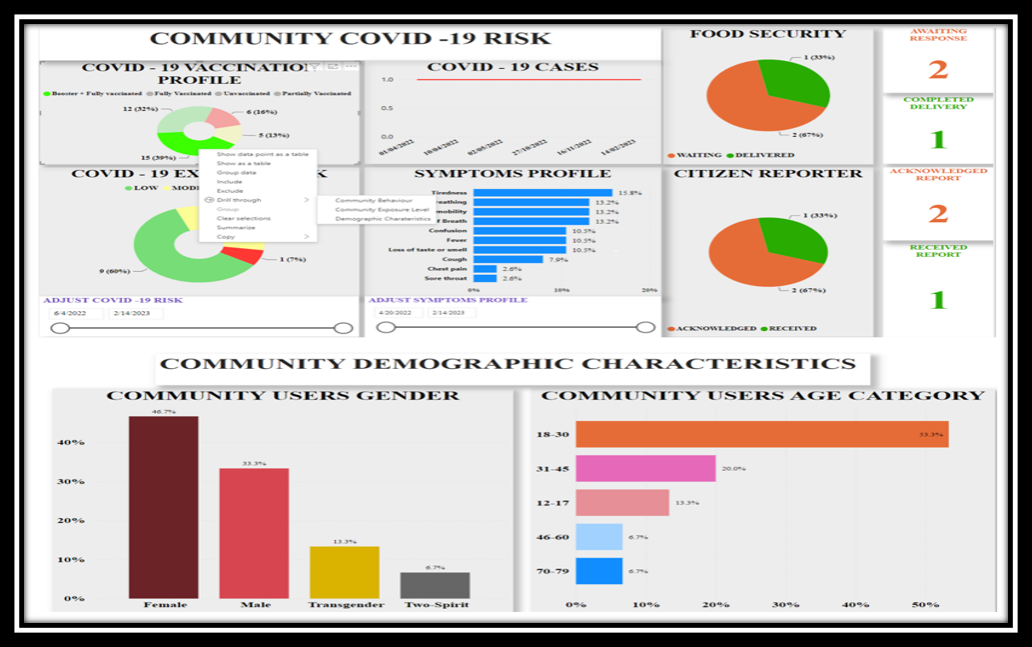
Data visualization depicting demographic characteristics along with community risks.

There is growing evidence that COVID-19–specific dashboards have played a significant role in pandemic management, particularly with regards to vaccine uptake to promote health equity [37]. The decision-making dashboard we developed provides detailed and anonymized aggregation symptoms, behaviors, and vaccine uptake status, which enabled decision makers to develop targeted public health communication in near real time to manage community risks. The data visualization of evolving and time-stamped statistics driven by real-time big data from the citizens of the community (Figure 9) provides a detailed picture of the changing nature of potential public health crises. For instance, it would be possible to understand the variation of prevalence and incidence of symptoms across specific time periods using dashboard functionality. This can be transformative not only for public policy but also for epidemiology in the digital age, where risks can be monitored in real time [51,52].

In enhancing the operational capacity of digital dashboards, particularly from the perspective of improving jurisdictional capacity, it is important to create delegated access to the dashboard. For instance, our digital dashboard has 7 different categories of access that enable the delegation of day-to-day tasks to ensure evidence-based decision-making across the jurisdictional operations. Moreover, the delegation of decision-making can even be outsourced to different operational sections within a municipal government or even outside the government. For instance, the food security feature could be delegated to a local food bank to ensure timely access to food. Perhaps more importantly, such delegated access enhances data security by providing permission-based restricted access to the dashboard.

In developing digital health dashboards for nonacademic users, particularly decision makers, it is important to prioritize intuitive visualization that does not require basic training in statistics or epidemiology. The data visualization within the dashboard is designed to be dynamic to make complex data sets easy to understand and explore by nonacademic audiences (ie, decision makers and public health practitioners), to extract information quickly, and enhance real-time rapid responses, particularly in the event of a public health crises. To ensure ease of uptake and use of the dashboard, we designed it to be interactive with the layering of data visualization, which allows decision makers to use intuitive tabs or dropdown menus to delve deeper to obtain the information they seek for evidence-based decision-making. For instance, if decision makers seek to obtain more information about community risk of COVID-19, such as distribution of vaccination status across age cohorts, or gender, they are able do this by selecting the available dashboard visualization options. Another key feature of the dashboard is depicting the temporality of data using data slicers, which allow decision makers to streamline evidence by specific time periods. For instance, data for a specific category of information (eg, COVID-19 vaccination) can be displayed over a specific time period, and compared with another category of information (eg, community symptoms) to understand how community behaviors correlate with general health of the community.

In terms of evidence-based decision-making in a world where there are new strains of COVID-19 as well as other evolving epidemics [53], the scalability and adaptability of the dashboard infrastructure are key to the sustainability of this innovation. Although this digital health dashboard was designed in response to the COVID-19 pandemic, this platform can be easily scaled up and adapted to monitor other epidemics and pandemics by using the developed digital infrastructure. The scalability is not limited to other epidemics or pandemics, but also across different levels of jurisdictions to ensure ethical monitoring, management, and mitigation of existing and emerging public health crises.

### Digital Citizen Science for Health Equity

Although citizen science is primarily considered a research approach that is participatory in nature and can range from contribution and collaboration with citizens to cocreation of knowledge [3], it is rarely used in the development of digital health platforms. However, the application of citizen science to public health decision-making processes has gained considerable momentum recently [54-58]. This potential and promise of citizen science informed our methodology for the development of our digital health dashboard. In developing this dashboard, we drew from the principles of the SMART Framework [12] that integrates citizen science, community-based participatory research, and systems science as well as the approaches of the global digital citizen science policy to tackle pandemics [3].

The DEPtH Lab’s Citizen Scientist Advisory Council enabled us to shape the dashboard to the needs of both the citizens and the decision makers [59]. The ability to balance both citizen and decision maker needs is critical to the success of digital health dashboards in providing a value perspective to both citizens at the frontend (PWA) and to the decision makers at the backend (digital health dashboard). The value perspective to the citizens is their ability to manage their household risks as well as understand community risks, which is inextricably linked to the functioning of the dashboard, which uses anonymized data derived from the PWA to understand and manage community risks along with the ability to respond to citizen needs in near real time.

Although the application of citizen science to public health is not new [60], this approach of citizen science for the development of digital health dashboards enables new opportunities to address public health crises from a systems perspective. For instance, the development of features to address food security and access to public services was based on the need identified by citizens, an approach that integrates health, food, and social service systems. The development of digital health dashboards using citizen science can potentially improve uptake from citizens as well as enhance scalability and adaptability across jurisdictions, that is, dashboards that are developed to address needs identified by citizens, an approach that will in turn meet decision maker goals in addressing citizen needs [59]. Thus, digital dashboards developed using citizen science can potentially transform public policy in multiple jurisdictions [61]. However, the biggest impact of these dashboards will be in promoting health equity by prioritizing and addressing the needs of citizens in near real time by developing equitable evidence-based policies using big data generated by the citizens themselves [12].

### Rapid Responses in Real Time

The real-time notifications feature of the digital health dashboard enables decision makers to engage with their community members effectively and directly to not only inform them about existing and emerging community risks but also to provide evidence-based public health communication. The big data received by the dashboard can be updated at intervals of 5 to 30 minutes to provide decision makers with recent and accurate community health information that can be used for rapid responses. From a public health management perspective, for instance, decision makers can inform community members of evidence of an increase in certain symptoms so that community members are aware of the changing dynamics of the health risks in their community.

However, the digital infrastructure of the dashboard can be repurposed based on the needs of jurisdictions, decision makers, or their citizens. Digital health dashboards can be paradigm changing in the age of climate emergency [62], where disaster management is directly linked with public health. For instance, currently, the digital dashboard is set up to send urgent alerts both with PWA and text alerts to inform citizens of all emergencies. This direct and rapid communication is critical for managing evolving risks that can range from pandemics and floods to forest fires and earthquakes [63,64].

Digital health dashboards can also be used to provide evidence-based public health information as well as policy and program information on a continuous basis. This feature can play an important role in minimizing misinformation and disinformation that is pervasive in this digital age [65]. The misinformation and disinformation related to public health [65], particularly in terms of pandemics, where false narratives about the disease and vaccines can cause considerable public health risks [66], can be minimized using digital health dashboards. The validity of the information can be cited or sourced using trusted external agencies such as the World Health Organization [67]. Nevertheless, it is difficult to address citizen mistrust in institutions, and the digital dashboards that are built using citizen science [3], and that operate using anonymized citizen data to generate evidence, can make incremental changes in citizen perception. Finally, the dashboard also provides a direct, bidirectional, and anonymized engagement with citizens that the citizens can initiate using the “Q & A” feature, which further enhances real-time engagement to build trust.

### Social Innovation: Decentralization of Technology and Data Sovereignty and Security

Digital health dashboards for decision-making that are developed for jurisdictions, at their very core, are operationalizing decentralizing of technology and democratization of data using digital citizen science [3,10]. This approach in essence is social innovation that ethically leverages the power of digital technology for decentralization and democratization to enable data sovereignty of individuals and jurisdictions. The decentralization is two-fold: (1) the development of a PWA that takes the platform ecosystem away from the monopolization of big technology launch platforms, that is, Google Play Store and Apple Store, and (2) the dashboard is set up to decentralize access to information, hence enhancing self-determination of individual jurisdictions. This decentralizing paves the way for the democratization of data, which are owned by citizens at the individual level and, by the jurisdictions, at the aggregated level [68].

This approach facilitates the data sovereignty of both citizens and jurisdictions, which is another important byproduct of the decentralization of technology. The decentralization of technology, democratization of data, and data sovereignty at both individual and jurisdictional levels provide the foundation for robust data security protocols. The design, implementation, and optimal functioning of digital health dashboards are dependent upon data privacy, and security. Apart from the standard measures of user authentication at both frontend (PWA) and backend (digital health dashboard) using multifactor authentication, data security protocols are enabled for both data at rest and data in transit in multiple layers. For data at rest, the first layer of security ensures restricted access to AWS account through identity and access management roles with multifactor authentication.

The second layer ensures that all data at rest are server encrypted using keys generated by AWS Key Management Service. The third layer ensures that all user database passwords are hashed and stored using Secure Hash Algorithm (SHA)-256 and SHA-512. For data in transit, to prevent distributed denial-of-service attacks, we use the Cloudflare-distributed denial-of-service mitigation service to ensure masking of website internet protocol address [69,70]. A distributed denial-of-service attack is a deliberate attempt to stymie a server, service, or network’s regular activity by saturating the target or its surrounding infrastructure with a deluge of internet traffic. To further ensure privacy and security of data in transit, Cloudflare server is used for key exchange and establishing the identity of a server by providing forward secrecy, and Advanced Encryption Server 128- Galois counter mode is used to encrypt the Hypertext Transfer Protocol Secure request. All responses are encrypted in the Transport Layered Security connection data and personally identifiable data are stored within user or citizen devices.

### Strengths and Limitations

A major strength of jurisdictional-specific digital health dashboards for decision-making is their potential to invert innovation [71]. Inverting innovation is defined as innovation that addresses a critical societal problem by satisfying 3 key criteria: transforming weaknesses into strengths, prioritizing citizen needs over corporate profits, and providing a pathway for rapid scale-up. Our digital health dashboard can respond rapidly to public health crises, by prioritizing both citizen and decision maker needs, and the digital infrastructure can be scaled-up based on evolving needs.

This inverting innovation potential of the digital health dashboard is driven by the digital citizen science approach [3], which is another major strength that ensures that the development process takes into consideration citizen needs. This focus on citizen needs also influenced our decision to prioritize decentralization of technology, democratization of data, and data sovereignty at both individual and jurisdictional levels—all aspects, which are significant strengths of the digital health dashboards. Finally, the primary purpose of development of jurisdictional-specific digital health dashboards is rapid responses using ethically obtained big data to monitor, mitigate, and manage population health crises, which is the most significant strength.

Even though rigorous data protection and security processes are layered into the dashboard development, risks to data security always exist, which need to be assessed on a consistent basis and addressed. Redundancies built into AWS databases allow us to protect the dashboard from ransomware attacks [72], and encryption and anonymization of data secure data privacy. Nevertheless, data privacy protocols need to be addressed on an ongoing basis to ensure that the dashboards comply with laws and regulations that govern jurisdictions [73]. Finally, citizen-driven digital health dashboards are limited by the uptake from citizens within jurisdictions, and the uptake is in turn influenced by internet equity [3] as well as digital divides within and across jurisdictions [74]. Although there is growing evidence of the potential of digital health dashboards to transform health care systems [15], the digital health dashboard that we developed needs to be tested in real-world settings. While the strengths and limitations of this study are specific to the development methodology and approaches, digital health dashboards powered by citizen-driven big data need to be tested in real-world settings to generate empirical evidence that can confirm not only the validity and veracity of citizen-driven big data but also the compliance of citizens and decision makers in using such dashboards for rapid decision-making.

### Conclusions

Digital health dashboards for decision-making can transform public health policy by prioritizing the needs of citizens as well as decision makers to enable rapid responses to public health crises. Digital health dashboards also provide decision makers the ability to directly communicate with community members to mitigate and manage emerging population health risks, a paradigm-changing approach to tackle future epidemics and pandemics, that is, inverting innovation by prioritizing community needs with digital technology. Finally, digital health dashboards that are built by using digital citizen science approaches can catalyze systems integration and pave the pathway for rapid scalability to ensure digital health for equity.

## Abbreviations

AWS: Amazon Web Services
DEPtH Lab: Digital Epidemiology and Population Health Laboratory
PHP: hypertext preprocessor
PWA: progressive web application
SHA: Secure Hash Algorithm

## Data Availability

The data generated by the progressive web application that are visualized in the digital health dashboard can be shared upon request.
